# Properties and Cementation Mechanism of Geopolymer Backfill Paste Incorporating Diverse Industrial Solid Wastes

**DOI:** 10.3390/ma16020480

**Published:** 2023-01-04

**Authors:** Haoyu Wang, Xianhui Zhao, Jing Wang, Lili He, Aijuan Zhang, Han Gao, Jing Yang, Luhui Liang

**Affiliations:** 1 School of Civil Engineering, Tianjin Renai College, Tianjin 301636, China; 2 School of Civil Engineering, Hebei University of Engineering, Handan 056038, China; 3 School of Mechanical Engineering, Tianjin Renai College, Tianjin 301636, China

**Keywords:** solid wastes, geopolymer, compressive strength, XRD, FTIR, microstructure

## Abstract

Industrialization has resulted in a large number of industrial waste slags being produced, which severely pollute the environment. This urgently needs resourceful treatment. The objective of this paper is to investigate the preparation, performance, and cementation mechanism of a novel geopolymer backfill paste for goaf. We reused diverse industrial waste slags based on low-calcium silica–alumina precursors (two fly ashes FAI, FAII, and red mud RM), high-calcium-based slags (carbide slag CS, soda residue SR, briquette residue slag BRS, and granulated blast furnace slag GBFS), and two additives (gypsum powder GP and lime powder LP). The hardening of backfill pastes was investigated by analyzing the effects of FAI, GBFS, RM, and LP on physical and chemical performance. The cementation mechanism of the prepared backfill paste was revealed through morphology, mineralogy, and chemical products through the use of X-ray diffraction (XRD), scanning electron microscopy (SEM), energy dispersive spectroscopy (EDS), and Fourier transform infrared spectroscopy (FTIR). The results show that the prepared backfill paste incorporating various solid wastes (FAI, FAII, RM, CS, SR, GBFS, RBS, etc.) yields a 28-d compressive strength of 2.1 MPa (higher than the required value of 0.6 MPa) and a fluidity of 201 mm. Geopolymer gels (N,C)-A-S-H, calcium silicate hydrated C-S-H, and calcium aluminosilicate hydrated C-A-S-H gels serve as chemical cementers, whereas unreacted particles serve as physical filler skeletons. These findings provide an experimental and theoretical basis for the interchangeable use of various identical component solid wastes in backfill engineering materials.

## 1. Introduction

Ordinary Portland cement (OPC) has been widely used as the most common cementitious material in the construction industry. However, the production process of OPC emits significant volumes of CO_2_ gas, posing a hazard to the environment [1,2]. It is estimated that 1000 kg of OPC synthesis results in about 950 kg of CO_2_ emissions [3]. Increasingly, strict government regulations now limit the manufacture and application of Portland cement in North China [3]. In addition, the treatment of industrial solid wastes, such as fly ash (FA) from thermal power plants and other slags from industrial enterprises, is also a severe social and environmental problem. Although a significant quantity of FA and slag have been reused in cement, concrete, and geotechnical materials, the remaining massive industrial solid wastes are mostly exposed to the open air, posing hazards to the local air, water, and land environments [4,5,6,7]. As an alternative to Portland cement, industrial wastes such as FA and slag can be used as much as possible in the research and development of new cementitious materials. This can help solve the two problems mentioned above. 

Geopolymers are made from FA, granulated blast furnace slag (GBFS), and other silica–alumina precursors activated by an alkaline solution [8,9,10,11]. Because of their extremely low waste emissions, low energy consumption, and outstanding mechanical performance, geopolymers are regarded as sustainable substitutes for Portland cement [12,13]. The geopolymer is usually made of strong alkali, which is strongly corrosive to the container. In addition, excessive alkali, which is more costly, can cause pan-alkali. Despite that, geopolymer has the potential to reduce CO_2_ emissions and energy consumption by approximately 80% and 60%, respectively [14,15]. In Australia, geopolymer materials made from industrial solid waste are used to substitute Portland cement materials, resulting in a 7% reduction in financial costs (without the cost of transportation distance) and a 44% reduction in CO_2_ emissions [16,17]. Moreover, the introduction of the Environmental Protection Tax Law (China) in 2018 has increased the disposal of industrial solid waste from some coal combustion and steel operations, among others, due to the 25-yuan tax collection per ton for improper solid waste disposal in companies [7]. As a result, geopolymer materials synthesized from industrial solid waste have been a hot research topic in recent years [18,19,20]. 

According to the empirical expression of M*_n_*[-(SiO_2_)*_z_*-(AlO_2_)-]*_n_* [4] for geopolymers, the common two-part geopolymers described were synthesized utilizing two combinations of silica–alumina solid precursors (Si-O and Al-O) and a strong alkaline solution (M) [21,22]. However, it is highlighted that during the synthesis and application of a two-part geopolymer, the strong corrosion feature of alkaline solutions as activators requires special attention and is supported with strict protective approaches. The restriction of the alkaline solution is extremely detrimental to the bulk synthesis and application of industrial waste based on geopolymer materials. As a result, in recent years, a one-part geopolymer has been introduced as a safe and convenient material that is manufactured by just adding water to solid binders [9,22,23]. One-part geopolymer paste is synthesized in the same way as traditional Portland cement slurry [9]. After the addition of water, the silica–alumina materials in a one-part geopolymer are alkali-activated by certain solid materials. Before being used, the silica–alumina materials (such as FA, red mud, slag, and so on) and solid activators were uniformly combined to make the one-part geopolymer binders [24,25]. Significant research efforts have been made in recent years to minimize the cost and alleviate the problem of strong alkali activation by exploiting various alkaline solid wastes as replacements for Portland cement to synthesize innovative binders [8].

Additionally, China has a variety of industrial solid wastes accessible, including low-calcium silica–alumina by-products and high-calcium-based waste slag. The typical low-calcium silica–alumina by-products include fly ash (FA) and red mud (RM), both of which have potential reactivities. Every year, over 500 million tons of FA is generally available from various enterprises in China [26,27]. The worldwide reservation of RM residues has reached an estimated dosage of 2.7 billion tons and is continually growing at a rate of 120 million tons per year [15,28]. Moreover, soda residue (SR), carbide slag (CS), and granulated blast furnace slag (GBFS) are types of high-calcium waste slag, which typically include 40% to 90% CaO. The SRs are produced by the industrial Na_2_CO_3_ process, and about 7.8–10.0 million tons of SRs is released yearly by more than 50 Chinese enterprises [6,7]. For every ton of PVC manufactured, about 1.5–1.9 tons of CS are generated. Moreover, the current CS reservation in China exceeds 10.0 million tons [29]. Therefore, based on local strategy and environmental protection, the growth emphasizes the importance of developing and implementing innovative methods of storage and remediation, as well as pursuing large-volume utilization alternatives for RM, FA, CS, and SR as harmful industrial by-products. 

The functionalities of FA, CS, BRS, SR, and GP have been disclosed in previous studies. Increasing the CS/GBFS ratio produces more crystal calcium hydroxide, which is detrimental to mechanical strength growth, whereas adding LP provides more Ca(OH)_2_ than CS, thereby accelerating GBFS hydration [2]. According to prior research, the incorporation of 20.0% CS into geopolymer composites based on FA lowered fluidity and enhanced long-term strength [5]. Due to their similar chemical components after combustion, BRS and CS should have comparable impacts on the properties of pastes. In addition, the necessary quantity of SR was added to FA-based geopolymer paste to improve the paste mixture’s characteristics for goaf backfill. The range study revealed that the fluidity and 28 d UCS increased and then decreased as the SR-FA ratio increased, with the greatest fluidity and strength occurring at an SR-FA ratio of 2:3 [7]. Then, 10% GP was substituted for Na_2_SiO_3_ in SR-FA-based geopolymer paste for goaf backfill, with a high fluidity of 196–222 mm and a suitable 28-d UCS of 0.92 MPa [30]. It was determined that 10% GP improved fluidity and UCS sufficiently to meet the performance criteria of goaf backfill material. In addition, Shi and Day [31] studied the effects of two FAs and lime on the development of the compressive strength and hydration of FA/GBFS combinations activated by NaOH and Na_2_SiO_3_ solutions. They also observed that the addition of a modest quantity of lime substantially enhanced the early UCS of alkali-activated materials. Consequently, each raw material used in this study has the chemical and physical properties to enhance the workability and mechanical strength of backfill pastes. In addition, it is necessary to determine the role of each raw ingredient to acquire the ideal mixing qualities of the backfill paste.

Moreover, some studies have been conducted to investigate the possibility of employing such industrial solid wastes as backfill materials through an alkali-activated technique [32]. High-calcium SR and CS were employed to improve the fresh and hardened performances of FA-based geopolymer paste. Through experimental programs, SR was combined with FA to make the paste for grouting backfill, and the optimal paste (SFN6) contains a high fluidity of 260 mm, a good compressive strength of 3.70 MPa, and a high stone ratio of 98.6% [7]. In addition, with long-term curing, CS was employed to improve the physical, mechanical, and microstructural properties of FA-based geopolymers [5]. Due to their similar chemical components, the reuse of CS was examined as a potential alternative to hydrated lime for the activation of GBFS [2]. Calcium-rich alumino–silicates, such as GBFS, are sometimes combined with low-reactive precursors, such as low-calcium FA, to promote system reactivity [27,33]. While the FA is partially replaced with GBFS, the key difference is the increased reactive CaO concentration, which has been identified as a beneficial component for improving mechanical strength in alkali-activated materials [34,35]. Additionally, owing to its high alkaline levels and high-quality aluminates, RM is thought to be suitable for geopolymer production. To fabricate geopolymer production, RM was combined with FA employing a composite activator [15,23,36]. The Ca(OH)_2_ and CaO are suitable alternatives to alkaline activators for the admixture or activator because their ingredients are significantly less expensive than NaOH and Na_2_SiO_3_ [30,37]. Currently, the commercialization of the alkali-activated binder is still a long way off due to the following challenges: (1) the development of new chemical admixtures; (2) the ensuring of workability, durability, and in-service track record; and (3) the lack of a detailed chemical understanding of the materials’ properties [12]. According to the foregoing studies, there is still a lack of knowledge of its underlying reaction mechanisms and principles, particularly for systems that comprise more than four wastes. Mine collapse prevention is a problem that cannot be ignored. The goaf area of mine tunnels usually needs to be filled with slurry to meet the requirements for strength. The use of industrial solid wastes to prepare backfill grouting materials has important advantages. However, there are many kinds of solid wastes, and whether similar components of solid wastes can be substituted for each other and how the cementation mechanism works require further research.

Hence, the major goal of this work is to reuse various solid wastes as green backfill materials. By adding water to diverse solid wastes (CS, SR, FA, RM, etc.), geopolymer backfill pastes were manufactured. The effects of FAI, GBFS, RM, and LP on the physical and chemical performances were investigated, including fluidity, compressive strength, pH, and EC values. Furthermore, the microstructure, products, and cementation mechanism were revealed by XRD, SEM-EDS, and FTIR analyses based on the optimal mixing ratio, offering references for using diverse solid wastes as engineering backfill materials. 

## 2. Experimental

### 2.1. Raw Materials

Raw materials such as low-calcium silica–alumina precursors, high-calcium-based slags, and additives are used to make geopolymer backfill pastes. Class I fly ash (FAI), class II fly ash (FAII), and red mud (RM) are the low-calcium silica–alumina precursors. Carbide slag (CS), soda residue (SR), granulated blast furnace slag (GBFS), and briquette residue slag (named BRS) are types of high-calcium-based slags. In addition, two additives, such as gypsum powder (abbreviated GP) and lime powder (named LP), are utilized. Diverse solid wastes in this work refer to solid combinations of FAI, FAII, RM, CS, SR, GBFS, and BRS generated from various industrial by-products. The chemical compositions and physical indices of these raw materials are given via X-ray fluorescence (Table 1).

(1) Low-calcium silica–alumina precursors

Class I fly ash (FAI): FAI is derived from a power plant in Gongyi, Henan Province, China. The FAI includes 2.8% CaO and is thus categorized as class F fly ash. The average particle size is 2.0 μm, with a maximum particle size of 10.0 μm. According to the Chinese standard GB/T 1596-2017, the sieved residual from 45.0 μm is less than 12% and the LOI is less than 5% for FAI. Class II fly ash (FAII): The FAII is derived from a power plant in Handan, China’s Hebei Province. FAII with 5.4% CaO is likewise categorized as class F fly ash. Additionally, the average particle size of 15.0 μm qualifies it as class II fly ash in terms of fineness (namely, the sieved residual of 45.0 μm is less than 30%, and the LOI is less than 8%, according to the GB/T 1596-2017 standard). FAII and FAI have similar chemical compositions except for Fe_2_O_3_, but FAII has much higher fineness and LOI than FAI. Red mud (RM): The RM is provided by an aluminum manufacturing enterprise in Gongyi, Henan Province, China. Because of their similar chemical compositions and potential reactivity, RM and FA were activated by the alkaline solution to form geopolymer material [23,36]. With higher alkalinity (Na_2_O), RM contains SiO_2_, Al_2_O_3_, and Fe_2_O_3_.

(2) High-calcium-based slags

To manufacture the backfill paste, four types of high-calcium-based slags (CS, SR, GBFS, and BRS) are used. The chosen CS is supplied by a production mode focused on the PVC manufacturing industry [5], which is supplied by a C_2_H_2_ chemical industrial plant in Qinghai, China. The CS includes 62.6% CaO and is categorized as category II industrial solid waste. The SR is obtained from a Na_2_CO_3_ production enterprise in Tangshan, China’s Hebei Province [6]. The SR contains up to 36.4% CaO and various soluble salts containing Cl^−^ and SO_4_^2−^. In addition, an S95-grade GBFS is also supported by Gongyi City, Henan Province, China. The GBFS contains 35.1% SiO_2_, 16.2% Al_2_O_3_, and 33.6% CaO. Although GBFS is considered an industrial by-product, it has a high reactivity to boost hydration [8]. The BRS is the residual slag of a civil briquette that is burned at 800–1400 °C, and the civil briquette encourages the development of cleaner energy as an alternative to original coal [38,39]. In chemical components, BRS has the same potential reactivity as CS. The BRS is provided by a coal plant in Handan, Hebei Province, China. The BRS is composed of SiO_2_ (14.7%), Al_2_O_3_ (1.2%), and 72.6% CaO.

(3) Two additives for backfill paste

The GP and LP are supplied by Xueli Building Material Company in Tianjin, China. Without any treatment, the GP and LP are utilized as additions to prepare the backfill paste. The GP (expressed as CaSO_4_·2H_2_O) has slight expansion properties [40], with 30.5% CaO and 40.6% SO_3_ by mass. Because of the high CaO content (71.2% by mass), the LP has high water absorption and hydration reactivity. Because Ca(OH)_2_ is a hydrated form of CaO, the significant strength difference of alkali-activated GBFS samples suggests that CaO may be a significantly more efficient activator than Ca(OH)_2_ [12].

(4) Cement material comparison

Ordinary Portland cement (OPC) #325 is utilized as a backfill paste comparison group. A construction material enterprise in Handan, Hebei Province, China, provides the OPC, which contains 65.3% CaO and 21.8% SiO_2_.

(5) Sodium hydroxide pellet and water

As a conventional alkali-activated material, the sodium hydroxide (NaOH) pellets used in the experiments were white particle reagents (analytical grade, purity ≥ 98%) considered an alkaline activator. A solid waste mixture mixed with partially granulated sodium hydroxide was used as a conventional alkali-activated comparison group binder. Kemiou Company in Tianjin, China, supplies the NaOH pellets. Furthermore, tap water with a pH of 7.40 and an electrical conductivity of 80.00 μS/cm is used, which is sourced from Handan in Hebei Province, China.

### 2.2. Mixing Design and Sample Preparation

Low-calcium silica–alumina precursors (FAI, FAII, and RM), high-calcium-based slags (CS, SR, GBFS, and BRS), two additives (GP and LP), and tap water were used to make the backfill grouting pastes. From the blending of raw materials to the demolding of hardened samples, the adopted preparation method is direct and convenient, as shown below. To achieve a homogenous mixture, all of the solid raw materials were blended in the designed mixing proportions, and then the required tap water was added according to the designed water–binder ratio (W/B). The obtained mixture was then mixed for three minutes at room temperature to synthesize fresh backfill paste by using a one-step mixing technique. The paste mixer (Model NJ-160A, Wuxi, China) has a stirring space of 62 ± 5 r/min and voltage/power (380 V/370 W). According to a previous study [7], the one-step mixing technique refers to the one-time addition of tap water into a solid mixture, similar to the preparation method of a one-part geopolymer [9,23]. After stirring, the fresh pastes were cast into the cylindric PVC molds (diameter of 36 mm and height of 72 mm) to cure under the conditions of 20 ± 2 °C temperature and 100% humidity. After curing for the required ages, the hardened paste samples were removed from the molds.

In addition, backfill pastes with good workability were used to characterize the fresh and hardened properties in comparison with OPC paste and alkali-activated paste made with NaOH. To achieve a comparison of performance, it was possible to synthesize materials that meet the requirements of the backfill engineering. After experiments, it was found that the water–cement ratio (W/C) of Q2 (OPC) was set at 0.64 to meet the requirements of fluidity and setting time. If the water–cement ratio is too high or too low, the fluidity and setting times are not applicable. Therefore, the water–cement ratio (W/C) for Q2 (OPC) is 0.64.

The backfill pastes were likewise made using the same mixing and curing processes as before. Table 2 shows the mixing proportions of the paste samples used (the ‘Q*’ group).

Furthermore, five paste groups (the ‘G*’ group) were designed to analyze the effects of FAI, LP, GBFS, and RM on physical and chemical performances and then to unveil the cementation mechanism of backfill paste, as shown in Table 2. The mixing and curing procedures were the same as the aforementioned method. The water contents in the mixing proportion were determined to improve the workability of fresh backfill pastes with an identical W/B of 0.70, which meets the fluidity criterion of goaf backfill engineering (See Table 3). After recording the temperatures before and after mixing, the fresh pastes were cast and cured in cylindric PVC molds. The samples were hardened and then processed with a H/D of 1:1 (36 mm in diameter and 36 mm in height) [7]. This proportion in Table 2 is a component that has been experimentally optimized for performance results based on the role of each solid waste component. Although the ratio is not unique, this ratio of components can meet the requirements for use in the goaf. In addition, the compositions of group G are identical to group Q. G5 is identical to Q1.

### 2.3. Testing Methods

#### 2.3.1. Determination of Fresh and Hardened Properties

Fresh properties of backfill pastes made from diverse solid wastes were determined, including temperature, pH value, electrical conductivity (EC), fluidity, and setting time. In addition, unconfined compressive strength (*UCS*) was determined. 

##### Measurement of Environmental pH and EC Values

The pH value reflects acidic and alkaline characteristics, whereas the EC demonstrates the presence of conductive soluble ions and cations [32]. The pH and EC of raw materials were determined after soaking solid raw materials weighing 20 g in tap water weighing 100 g (a solid-to-water ratio of 1:5) for 24 h in glass containers. Moreover, the glass containers were sealed at room temperature to prevent water loss. In addition, the pH and EC of the environmental liquids were evaluated when the hardened pastes were soaked in water to analyze the impact of hardened pastes as goaf backfill on environmental water. The tests were carried out after 30 g of hardened samples were soaked in 150 g of tap water (a solid–water ratio of 1:5) for 1, 7, and 28 days at room temperature. A DDS-307A conductivity meter with an electrode probe was used, with a resolution of 0.01 mS/cm, a maximum range of 100 mS/cm, and an electrode probe constant of 10 cm^−1^. To guarantee the reproducibility of the results, at least five separate measurements were taken. 

##### Measurement of Fluidity and Setting Times

The flow diameters of fresh backfill pastes were measured to assess fluidity following ASTM C230/C230 M. After three minutes of stirring the solids and tap water, the freshly synthesized pastes were cast into a slump cone mold (top diameter: 36 mm, bottom diameter: 60 mm, and height: 60 mm) on a glass plate [7,22,23]. After scraping the paste off the top surface with a steel ruler, the slump cone mold was withdrawn vertically. After that, the fresh paste flowed freely for 30 s while the steel ruler measured the two maximum diameters in the vertical direction. The fluidity of fresh paste was determined using the average of two maximum diameters. From the addition of water to the determination of flow diameter, the fluidity testing was accomplished in six minutes. 

Setting time, one of the most important properties of binder paste materials, is used to characterize the time when the backfill paste begins to lose plasticity (referred to as the initial setting time) and another time when plasticity completely vanishes and strength begins to form (referred to as the final setting time) [41]. The Vicat apparatus was used to measure the initial time and final setting time following GB/T 1346-2011 (China).

##### Determination of UCS

The *UCS* is a major mechanical performance of a hardened backfill paste that is utilized to determine if the backfill paste is damaged when subjected to external loading [42]. At 7 days, the hardening of the paste and controls, such as G1, G2, G3, G4, and G5, was first observed. An unconfined compressive strength machine with a loading strain rate of 1.0 mm/min was used to test the UCS of hardened pastes and controls at 28 and 90 days [43]. The findings were calculated using the mean of five identical hardened samples. The following Equation (1) was used to obtain the UCS (MPa) result:

UCS = *M*_L_/*A*_0_ (1)

where *M*_L_ denotes the maximum load at failure (N), and *A*_0_ denotes the average bed face area (mm^2^).

#### 2.3.2. Microcharacterization Tests of Hardened Pastes

Following the determination of fresh and hardened properties, the XRD, SEM-EDS, and FTIR tests were conducted to examine the microcharacteristics and reveal the cementation mechanism of backfill paste G5 with diverse solid wastes compared to that of raw materials. 

The XRD patterns were acquired to characterize the crystal and amorphous phases in backfill pastes and controls [8,37]. Table 2 shows the XRD samples obtained from raw materials and synthesized samples at 90 days. With a scanning rate of 2°/min and a scanning range of 10°~80° 2θ, X-ray diffraction spectroscopy (XRD, Rigaku D/MAX-2500, Japan) was used to explore the mineral phases of 90 d age.

The SEM-EDS tests were performed to examine the morphology, microstructure, and elemental components of 90 d samples (Table 2) compared to raw materials. To investigate the main elemental distributions for different phases, the EDS spectra were acquired using a mapping technique. A scanning electron microscope (SEM-EDS, Quanta FEG450, Hillsboro, OR, USA) with energy dispersive spectroscopy was employed.

The FTIR spectra were obtained to further examine the product changes from the characteristic vibration peaks of chemical bonds [44]. In Table 2, the FTIR tests were performed on 90 d samples in comparison to raw materials. The samples were air-dried for 48 h before being crushed into powders. The tested specimens were then prepared by combining the powders (1.3 ± 0.001 mg) with potassium bromide (KBr) pellets (weighing 130 mg). The test was performed using Fourier transformation infrared spectroscopy (FTIR, Nexus 8, Bruker, Karlsruhe, Germany) with a wavenumber range of 500~4000 cm^−1^ (accuracy of 1 cm^−1^).

## 3. Results and Discussion

### 3.1. Performance Assessment of Geopolymer Paste Incorporating Diverse Solid Wastes

According to the experimental results, the mixing proportion of paste G5 (namely Q1) is 7.14% FAI, 28.57% FAII, 7.14% RM, 14.29% CS, 7.14% SR, 7.14% GBFS, 7.14% BRS, 7.14% GP, and 14.29% LP by mass, with a water–binder ratio of 0.70 and a density of 1500 kg/m^3^. In terms of basic performance, backfill paste Q1 has a fluidity of 201 mm, and its initial setting time, final setting time, and 28 d *UCS* meet the property requirements of backfill paste as an alternative to OPC paste Q2, as shown in Table 3. In addition, as a comparison sample, solid NaOH pellets were incorporated into the backfill paste in sample Q3. In comparison to sample Q3, Q1 has superior workability but a similar 28 d *UCS*, and the initial and final setting times are shorter. Then, in terms of environmental pH and EC values, Q1 has a lower environmental impact than Q3 and Q2. Hence, the properties of G5 meet the basic engineering requirements for goaf backfill.

The research objective of the new backfill materials is to achieve a one-part mixing method and one-step mixing technique by directly adding water, to realize the substitution of diverse industrial solid wastes, and to control the fresh and hardened properties such as fluidity, setting time, UCS, and stability. From the experimental results, it is concluded that geopolymer paste made from various solid wastes (accounting for 40–60%, and containing cheap additives) produces good self-hardening for goaf backfill. Furthermore, the fluidity, 28 d UCS, setting time, etc., of the new backfill paste are comparable to those of OPC paste, with better stability in harsh environments.

To reduce transportation costs resulting from transportation distance, diverse solid waste should be taken from waste resources in the urban areas adjacent to the current application works, as well as adjacent provincial and municipal areas, as soon as possible. In addition, although the particle size of raw materials used as the grouting slurry is usually not strictly required, the particle sizes influence the chemical reaction activity of backfill paste, thus affecting the flow, setting time, and compressive strength. However, the particle sizes of materials do not affect the nature of the chemical reactions between diverse solid wastes.

### 3.2. Effects of FAI, LP, GBFS, and RM on Physical and CHEMICAL Performances

**Figure 1:**
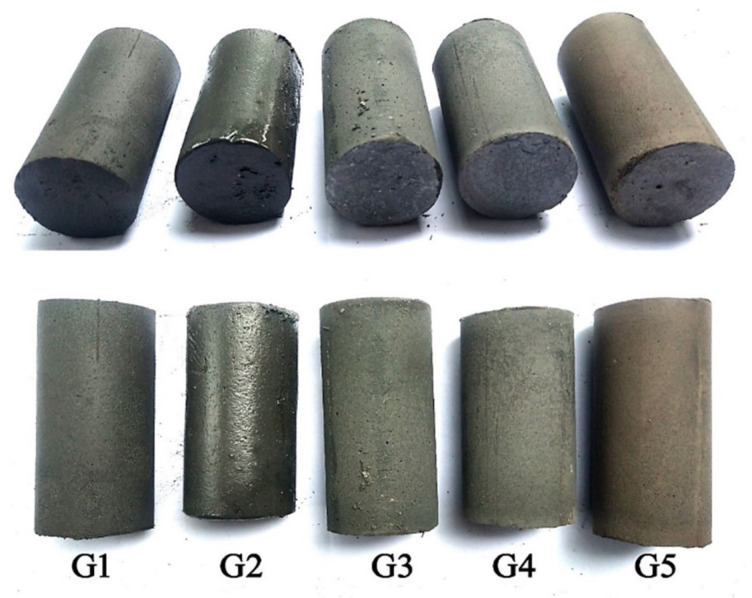
The hardening observation of paste (G5) and controls (G1, G2, G3, and G4) at the curing age of 7 days.

After the paste Q1 (namely G5) was synthesized and characterized, the cementation mechanism was further clarified by combining physical and chemical properties with microstructural characteristics and product compositions. According to the designed method in Table 2, the FAI, LP, GBFS, and RM were added to the mixture one by one to elaborate on the variations in reaction processes and products, as well as to investigate the effects of raw materials in the backfill paste. All samples (G1, G2, G3, G4, and G5) were removed from molds after 7 days of hardening to evaluate the hardening condition and verify the mutual reaction, as shown in Figure 1. The measured physical and chemical performances of the pastes are shown in Table 4.

As with G1, the paste of CS, SR, BCS, FAII, GP, and water yields an unsatisfactory hardening with low *UCS*. Similarly, G2 has a poor hardness at 7 d like G1 (Figure 1). This explains why the 28 d and 90 d *UCS*s remain less than 0.2 MPa for G1 and G2 until 28 d (Table 4). However, the pastes of G3, G4, and G5 exhibit acceptable hardening at 7 d (Figure 1), which corresponds to the 28 d and 90 d *UCS*s in Table 4. Correspondingly, based on the ph value of fresh paste, the specimens with a ph value lower than 9.24 harden poorly, and those with a ph value higher than 12.23 harden better. All raw materials were blended with water to produce an exothermic reaction, as evidenced by the increasing temperature of 0.5–1.5 °C before and after mixing for two minutes (Table 4).

Due to the poor cementation derived from the extremely low 28 day and 90 day *UCS*s, the fluidity of the mixture increases with the addition of FAI, but the pH and EC of the mixture decrease. In G1, high-calcium-based slags such as CS, SR, and BRS have a slow hydrated reaction with low-calcium FAII [5,7,37], and it is not obvious for GP, as an addition, to promote faster hydration to achieve better strength in a higher W/B of paste [30]. Subsequently, the nature of the hydration in G2 does not alter when low-calcium FAI is added to mixture G1. Hence, G2 maintains extremely low 28 d and 90 d *UCS*s but enhances fluidity due to the rolling function and low reactivity of the glass microspheres in FAI. 

The inclusion of LP increases the 28 d *UCS*, the 90 d *UCS*, and the pH value, but decreases the EC and the fluidity. The higher hydration rate occurs in G3 due to the inclusion of CaO in LP [45]. The Ca(OH)_2_ is formed when CaO reacts with water, reducing fluidity and increasing the alkaline environment.

Furthermore, the inclusion of GBFS results in an increased fluidity, an increased 28 d *UCS*, an increased 90 d *UCS*, and an increased pH value but a decreased EC. The hydrated and geopolymeric processes in G4 are accelerated at a pH of 12.31. Under these conditions, the dissolution of SiO_2_ and Al_2_O_3_ was accelerated, allowing them to react with CaO to form hydration products such as C-A-S-H and C-S-H gels [19,45].

When RM is introduced, the 28 d and 90 d *UCS*s are dropped to 2.1 MPa and 3.4 MPa, respectively, but the fluidity and pH are increased, and the EC is decreased. RM possesses geopolymerization reactivity, and its addition easily promotes the highly alkaline environment and the dissolution of SiO_2_ and Al_2_O_3_ to generate geopolymer gels [15]. However, with alkaline activation, the reactivity of RM is lower than that of GBFS [46]. As a result, the *UCS* of G5 is lower than that of G4. Except for G3 and G4, the addition of RM in G5 increases the *UCS*s from 28 to 90 days due to the continuous geopolymeric and hydration processes. Therefore, the effects of FAI, LP, GBFS, and RM are clearly stated in the physical and chemical properties of backfill pastes. The results are consistent with the hardening of samples shown in Figure 1. 

### 3.3. Mineral Phase Analysis by XRD

#### 3.3.1. Mineralogy Analysis of Raw Materials

**Figure 2:**
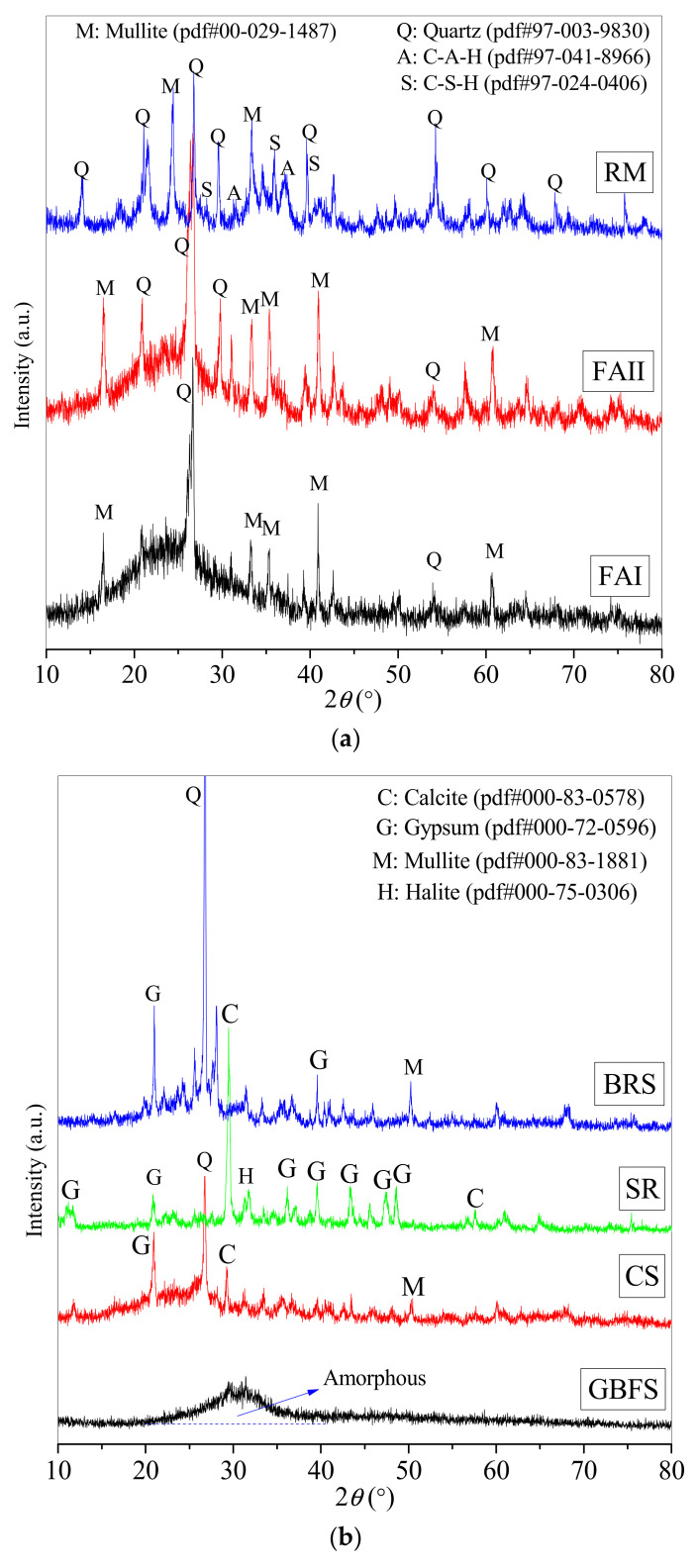

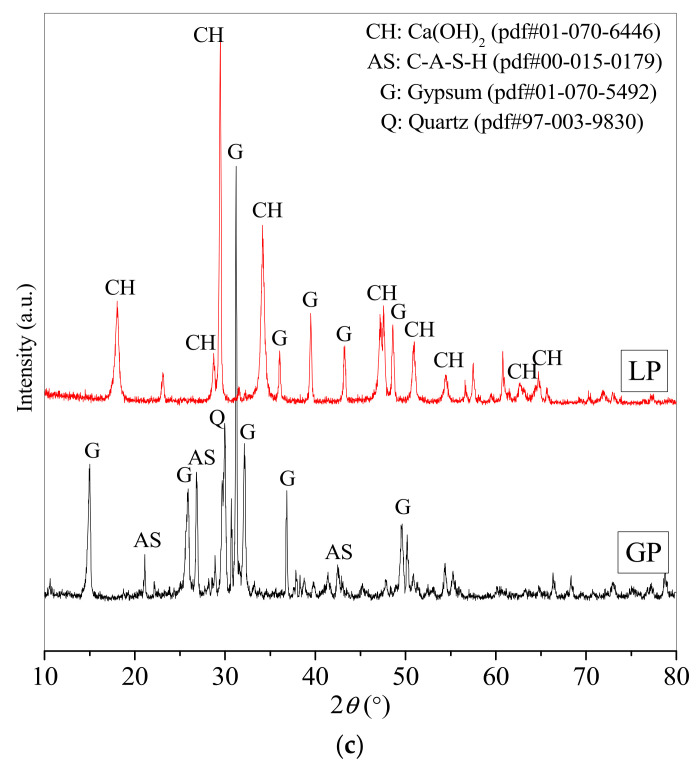
The XRD patterns of raw materials: (**a**) low-calcium silica–alumina precursors (FAI, FAII, and RM), (**b**) high-calcium-based slags (CS, SR, GBFS, and BRS), and (**c**) two additives (LP and GP).

**Figure 3:**
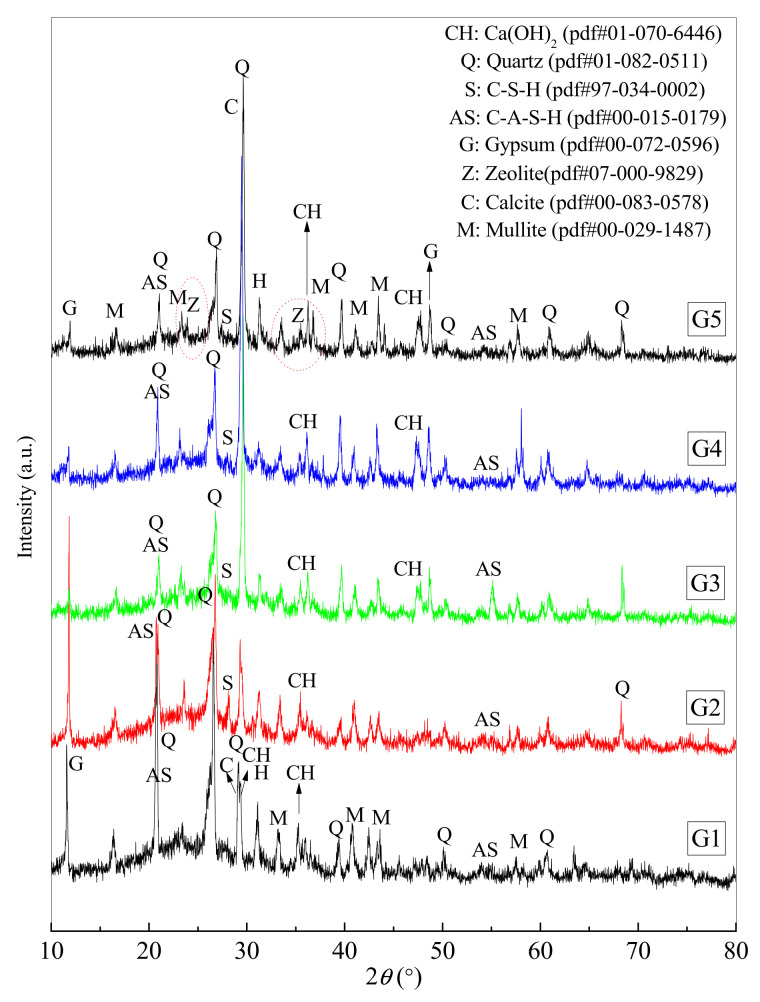
The XRD patterns of backfill paste (G5) and controls (G1, G2, G3, and G4) at 90 days.

The crystal and amorphous states of gel products directly present the compositions and microstructural properties of the materials [47]. Figure 2 and Figure 3 give the XRD patterns of raw materials as well as a paste (G5) and controls (G1, G2, G3, and G4) at 90 days.

As for the low-calcium silica–alumina precursors in Figure 2a, the broad humps appear in different angle ranges (FAI, 15–40° 2θ; FAII, 15–40° 2θ; and RM, 30–40° 2θ) due to the existence of amorphous silica–alumina phases [15,23,48]. It can be seen that FAI and FAII contain quartz and mullite crystal compositions, while RM contains C-A-H and C-S-H crystal compositions in addition to quartz and mullite. The early self-hydration reactions of CaO, SiO_2_, and Al_2_O_3_ produce some crystalline C-A-H and C-S-H in RM [49]. 

In terms of the high-calcium-based slags in Figure 2b, the XRD pattern of GBFS shows a broad hump at 20–40° 2θ, which is attributed to a large number of amorphous silica and alumina phases [8,48], but no crystalline phases are detected, which is consistent with the results in the references [33,35]. In addition, due to the industrial combustion process, CS and BRS also contain a small amount of amorphous silica–alumina phases. Moreover, the crystal phases in CS are mainly gypsum, quartz, calcite, and mullite, while the crystal phases in BRS are mainly gypsum, quartz, and mullite without calcite. The crystal differences in CS and BRS can be seen to be crystal calcite. Additionally, SR contains crystals of gypsum, quartz, halite, and calcite, which agree with the mineral compositions of previous research [21,37]. 

The XRD patterns of the additives LP and GP in Figure 2c show significant crystalline diffraction peaks but no broad hump for the amorphous phase, which is consistent with previous results [12,30,45]. It can be seen that LP has Ca(OH)_2_ and gypsum as its main crystal compositions, whilst GP has gypsum, quartz, and C-A-S-H as its main crystal compositions. 

Mineral analyses from XRD patterns in Figure 2a–c contribute to a better understanding of the properties and compositions of raw materials and encourage the further chemical synthesis of the backfill paste by using a variety of industrial solid wastes.

#### 3.3.2. Mineralogy Analysis of Pastes

Figure 3 shows the XRD patterns of paste G5 and controls (G1, G2, G3, and G4) at 90 d. The bad hardenings of G1 and G2 occur at pH values of 9.24 and 9.15, respectively, from the aforementioned 28 d and 90 d UCSs, indicating low hydration, and thus the broad humps of G1 and G2 are mainly the superposition of all amorphous humps in FAI, FAII, BRS, and CS. The additions of LP, GBFS, and RM improve the hardening of G3, G4, and G5 at a pH value higher than 12.23. The incorporation of amorphous SiO_2_ and Al_2_O_3_, as well as the generation of more amorphous products, affects the intensity and angle center of broad humps. Because the XRD patterns do not show the specific amorphous compositions of cementitious products identified as C-S-H or (N,C)-A-S-H gels, etc., other testing techniques are required. 

In terms of crystal phases, the gypsum phase has a high intensity in G1 and G2, but the additions of LP, GBFS, and RM cause the intensity of gypsum to decrease at 12° 2θ. This could be due to the gypsum phase being involved in the chemical reactions, or it could be due to the gypsum content being reduced as well as wrapped by the formed amorphous product gels [13]. According to the detected XRD patterns, the G3, G4, and G5 contain the crystal compositions of Ca(OH)_2_, gypsum, mullite, quartz, calcite, halite, C-A-S-H, C-S-H, and zeolite. A high peak at 29–30° 2θ accounts for the poorly crystalline C-S-H [50,51], and C-A-S-H is found at the reflection of 2 = 54–56° 2θ and is associated with the poorly crystalline C-S-H and C-A-S-H [2]. The higher intensity of C-A-S-H at the reflection of 2 = 54–56° 2θ in G3 accounts for the addition of LP due to the promotion of a higher hydration degree to form C-A-S-H. G5 contains crystal zeolite, which is distinct from G4 and G3. According to the crystal zeolite formation process, aluminosilicate gels such as N-A-S-H are formed by the dissolution and geopolymerization of amorphous SiO_2_ and Al_2_O_3_ in an alkaline environment, and then the aluminosilicate gels crystallize to be zeolite under the proper conditions. Aluminosilicate gels are considered to be the precursors of crystal zeolite [52]. As a result, XRD patterns indicate that the mixture of C-S-H, C-A-S-H, and geopolymeric gels in 90 d products for backfill paste G5 and other crystal phases are derived from raw materials. However, the specific amorphous phases should be determined further.

### 3.4. Morphology and Microstructure Analysis by SEM-EDS

#### 3.4.1. Morphology Analysis of Raw Materials

**Figure 4:**
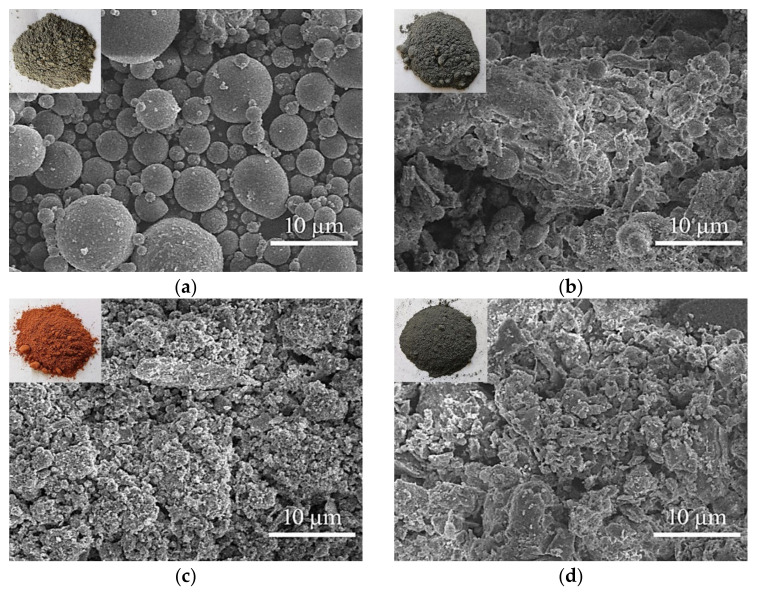

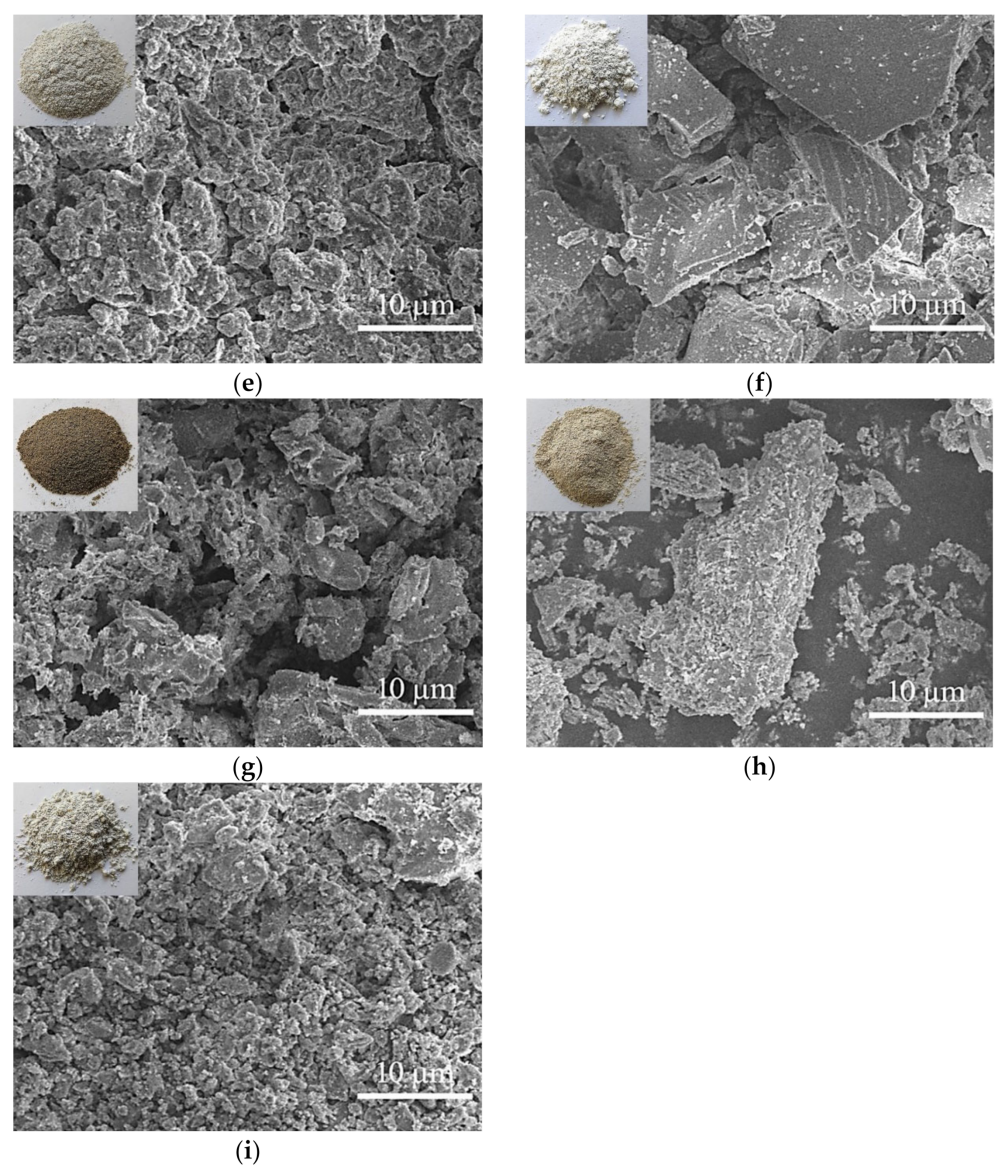
The SEM images of raw materials: (**a**) FAI, (**b**) FAII, (**c**) RM, (**d**) CS, (**e**) SR, (**f**) GBFS, (**g**) BRS, (**h**) GP, and (**i**) LP.

The morphologies of raw materials were examined using SEM images (Figure 4). FAII has spherical glass beads and irregular particles for the main morphology, which are similar to FAI’s spherical glass beads. The main glass beads are formed by rapid cooling after high-temperature burning [4,29]. RM, CS, GBFS, BRS, and LP exhibit particle morphology such as flaky, blocky, and others, all of which exhibit dispersed structural characteristics [34,35,36,53], while the GP exhibits agglomerated structure. In addition, SR contains spherical, flaky, and cloudy particles and has an agglomerated and loose structure. Spherical and granular substances, in particular, improve the fluidity of backfill pastes before they are dissolved [30,54,55]. In addition, under alkaline conditions, all raw materials show potential reactivity [56,57]. FAI, FAII, RM, and GBFS exhibit potential reactivity to alkaline hydration and geopolymerization processes. The hydration is determined by the chemical reaction of CaO, Ca(OH)_2_, and silica–alumina components to form layered C-S-H, C-A-H, and C-A-S-H gels [47]. Geopolymerization, however, results from a chemical polymeric reaction between Al-O and Si-O bonds coupled with the cations Na^+^ and Ca^2+^, etc., to produce the main sodium aluminosilicate gel (N-A-S-H) and calcium-containing sodium aluminosilicate gel (N,C)-A-S-H [4,13,47]. In general, the glassy SiO_2_ and Al_2_O_3_ in FAI, FAII, RM, and GBFS were dissolved and then geopolymerized to form aluminosilicate polymeric gels [45,55,58]. GBFS also serves as the main calcium source in chemical compositions. Others, mainly calcium sources, such as GP, LP, SR, CS, and BRS, exhibit potential reactivity under hydration due to the efficient chemical components CaO and Ca(OH)_2_. Thus, following hydration and geopolymerization, the formed product gels, which are produced from raw materials, provide chemical cementation between particles [8,19,45].

#### 3.4.2. Morphology and Microstructure Analysis of Hardened Pastes

**Figure 5:**
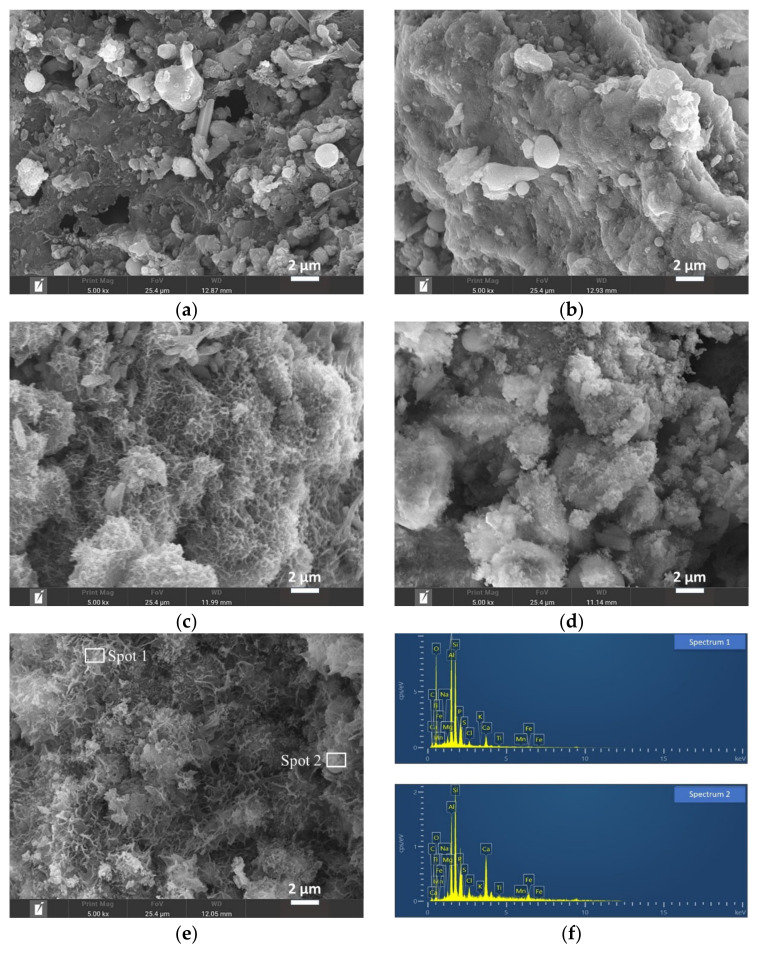
The SEM images and EDS spectra of paste (G5) and controls (G1, G2, G3, and G4) at 90 d: (**a**) SEM of G1, (**b**) SEM of G2, (**c**) SEM of G3, (**d**) SEM of G4, (**e**) SEM of G5, and (**f**) EDS spectra of spot 1 and spot 2.

Figure 5 shows SEM images of G1, G2, G3, G4, and G5. The exposed more granular substances in G1 and G2 were far less cemented (Figure 5a,b). The unreactive particles in G3, G4, and G5 were wrapped in the more cemented substance with flocculent (Figure 5c–e). Additionally, the flocculent morphology of G3 and G5 is the same, so the products may be the same. The EDS spot test was performed on the flocculent cementation product from G5 (Figure 5e), and the results were found to be similar for both spots (spot 1 and spot 2), with O, Si, Al, and Ca elements predominating, excluding a small number of elemental impurities such as Fe and Cl (Figure 5f). Therefore, the cementation product is most likely a C-S-H and/or C-A-S-H gel. This is the product of the hydration reaction of G3 lime powder mixed with silica–alumina precursors.

The products were characterized by combined XRD, SEM-EDS, and FTIR. Since the main elements are O, Si, Al, and Ca, the hydration product gels may only be C-S-H, C-A-H, or C-A-S-H. Combined with the results of the XRD mineral analysis (Figure 3 in the revised manuscript), the product C-A-H can be excluded. Therefore, the products should be C-S-H or/and C-A-S-H.

**Figure 6:**
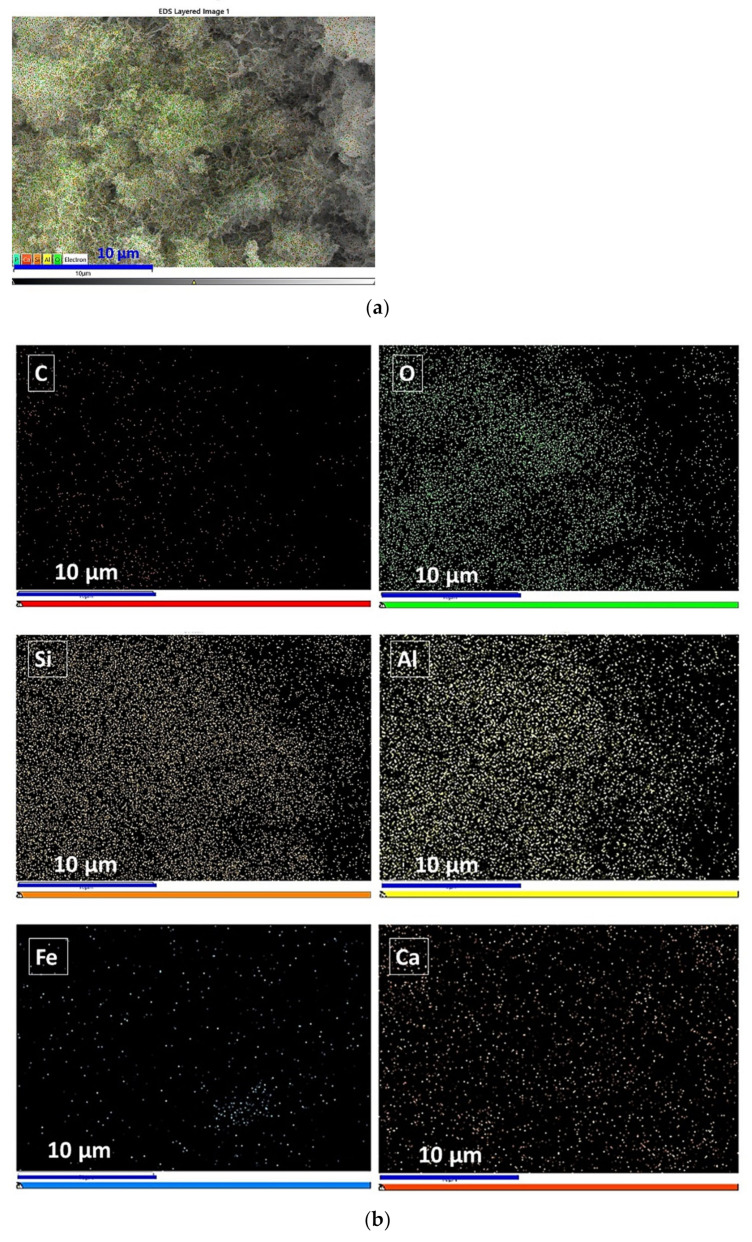

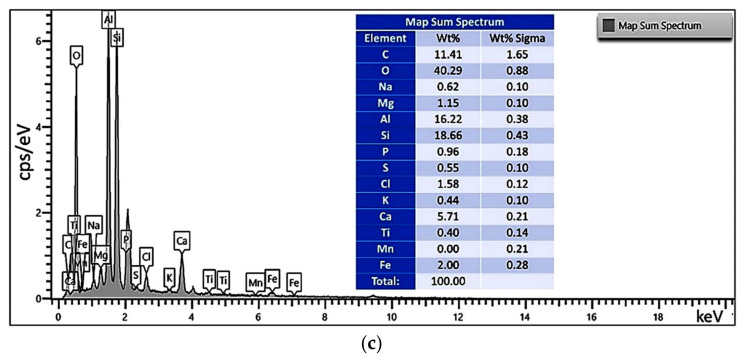
The EDS spectrum and elemental distribution of paste (G5) at 90 d by mapping technique: (**a**) EDS mapping, (**b**) elemental distribution, and (**c**) EDS spectrum.

As shown in Figure 6, the EDS mapping was used to observe the distribution of the main elements in G5. C, O, Si, Al, Ca, and Fe predominated in the overall interfacial element distribution chosen (Figure 6a,b). The small proportion of Fe elements and their uneven distribution in Figure 6c are caused by the incomplete dissolution of the RM particles. Carbonates from the SR and carbonates formed during the curing process are unevenly distributed and mostly concentrated in the lower left position of the interface according to the C element. After the high-temperature calcination of silica–alumina phases to be the precursors such as FA, GBFS, and RM, the amorphous SiO_2_ and Al_2_O_3_ (main elements O, Si, and Al) are formed. The three elements are more sparsely distributed in the upper right part and denser in the lower left part, indicating that there are still no fully dissolved silica–alumina phase particles. Although G5 has low reactivity, the final products are C-S-H and C-A-S-H. The more uniform distribution of Ca elements indicates that the added lime powder and the calcium source derived from the raw material are highly dissolved and distributed evenly in the product gel. Otherwise, the Ca elements would clump together. In summary, in an alkaline environment, some of the silica–alumina precursors dissolve and combine mainly with the calcium source to form flocculent C-S-H and C-A-S-H gels. The gel wraps the unreacted particles, and then solidification and coagulation occur, resulting in the solidification of the grouting material. 

### 3.5. Product and Chemical Bond Analysis by FTIR

**Figure 7:**
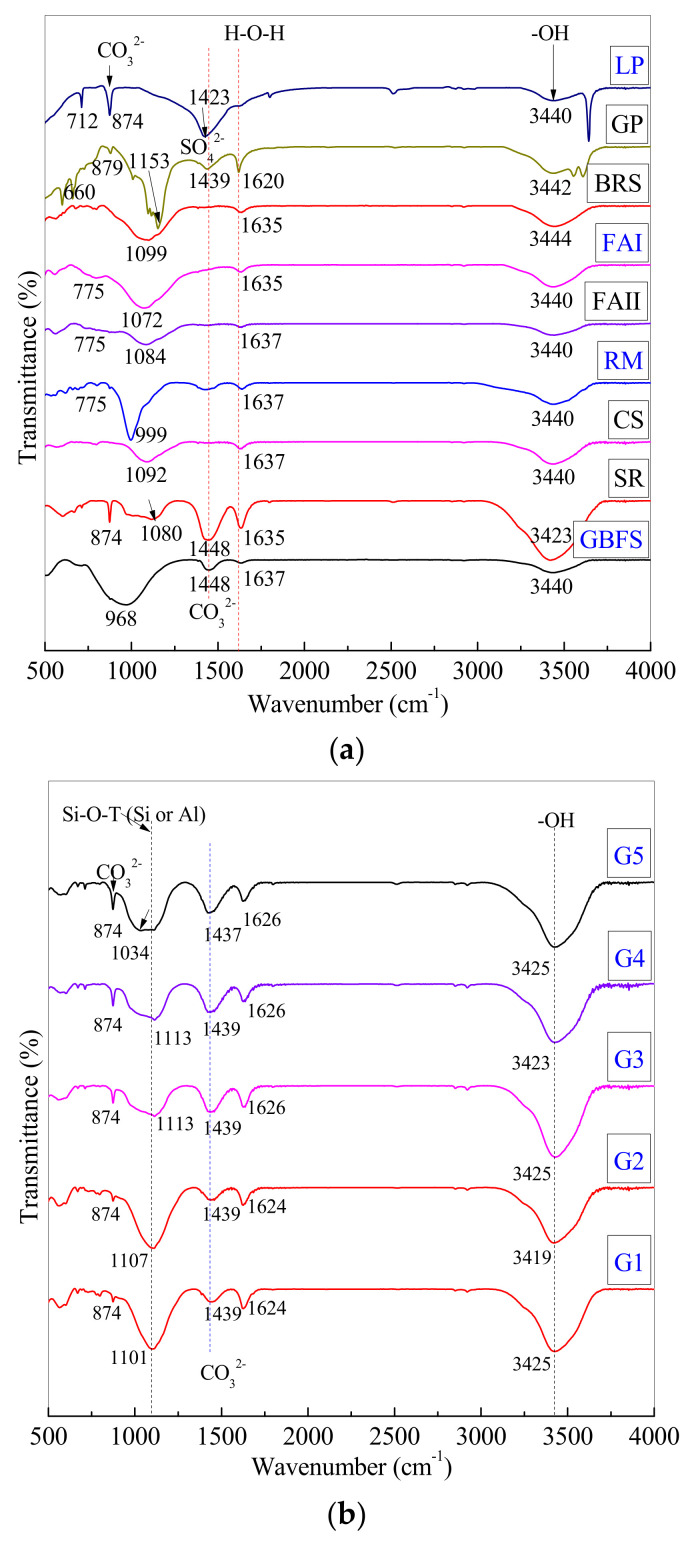
The FTIR spectra of (**a**) raw materials and (**b**) pastes G1, G2, G3, G4, and G5 at 90 d.

Figure 7 shows the FTIR spectra of backfill paste (G5) and controls (G1, G2, G3, and G4) in comparison to raw material spectra. Table 5 shows the specific wavenumbers corresponding to the characteristic absorption peaks. The stretching vibration of the -OH bonds from H_2_O is attributed to the absorption peaks at 3419, 3423, 3425, 3440, 3442, and 3444 cm^−1^ [30,59], whereas the bending vibration of the H-O-H bonds from H_2_O is attributed to the absorption peaks at 1620, 1624, 1626, 1635, and 1637 cm^−1^ [6,60]. These absorption peaks demonstrate the presence of moisture in partially oven-dried materials during specimen preparation. The raw materials FAI, FAII, BRS, CS, GBFS, and RM contain Al-O-Si chains, which are attributed to the absorption peaks at 960–1100 cm^−1^ from the silica–alumina phase and have the potential reactivity to dissolve in alkaline environments [61,62]. This is also because these raw materials are produced through high-temperature combustion. However, LP, GP, and SR contain calcium sources such as CaCO_3_ and CaSO_4_ at peaks of 1423, 1439, and 1448 cm^−1^ [8,63], as well as CaCO_3_ at peaks of 874 and 879 cm^−1^ [8,64]. Therefore, the raw materials support both the potential silica–alumina phase and the calcium source for backfill paste preparation, based on FTIR spectra.

FTIR spectra reflect various chemical bonds in materials. Although there are various types of silica–aluminum phase bonds, this paper focuses on the peak shift (change in wavenumber of absorption vibration peaks) of the characteristic peaks before and after the reaction. The shift in peak position can only occur for substances that have undergone chemical reactions. The results of the FTIR spectra and the XRD diffraction spectra have complementary descriptions to each other. In addition, the XRD, SEM-EDS, and FTIR methods used are combined to characterize the products, as determined by the qualitative methods employed. The FTIR analysis results are also within the accuracy of the tests.

**Table 5:** The absorption peaks of related chemical bonds by FTIR spectra.

Chemical Bonds	Vibration Styles	Wavenumbers (cm^−1^)	Sources/Substances
-OH	Stretching vibration [30,59]	3419, 3423, 3425, 3440, 3442, 3444	H_2_O
H-O-H	Bending vibration [6,60]	1620, 1624, 1626, 1635, 1637	H_2_O
CO_3_^2−^	Asymmetric stretching Vibration [8,63]	1448, 1439, 1437	CaCO_3_
SO_4_^2−^	Asymmetric stretching Vibration [8,63]	1423	CaSO_4_
CO_3_^2−^	Bending vibration [8,64]	874, 879	CaCO_3_
Si-O-T (Si or Al)	Asymmetric stretching vibration [4,7,21]	1153, 1099, 1072, 1084, 999, 1092, 1080, 968, 1034, 1113, 1107, 1101	C-S-H and (N,C)-A-S-H
Si-O or Al-O	Bending vibration [61,62]	775	SiO_2_ and Al_2_O_3_ in precursors

Paste G1 is made by combining BRS, FAII, CS, SR, and GP with water. It is discovered that the formed products with Si-O-Si chains correspond to the absorption peak at 1101 cm^−1^ and that some calcium silicate hydrated (C-S-H) gels form in G1, which is consistent with the aforementioned XRD results. The wavenumber at 1101 cm^−1^ in G1 shifts to a slightly higher wavenumber at 1107 cm^−1^ in G2. Due to the addition of FAI, the silica–alumina phases increase and then promote a reaction with the calcium source to form the hydration product C-S-H in water environments. Because of the small amount of formed C-S-H and C-A-S-H gels, G1 and G2 have lower cementation strengths (Table 4). As a result, the addition of FAI only increases the reactants of the silica–alumina phases in G1 and G2, without significantly increasing cementation. The chemical reactions are represented by the Formulas (2) and (3) reported in previous studies [21,37]. The reaction parameters are *x*, *y*, and *n*.

*x*Ca(OH)_2_ + SiO_2_ + (*n* − *x*)H_2_O → *x*CaO·SiO_2_·*n*H_2_O [C-S-H] (2)

*x*Ca(OH)_2_ + *y*Al_2_O_3_ + SiO_2_+ (*n* − *x*)H_2_O → *x*CaO·*y*Al_2_O_3_·SiO_2_·*n*H_2_O [C-A-S-H] (3)

Additionally, the wavenumber at 1107 cm^−1^ in G2 shifts to a slightly higher wavenumber at 1113 cm^−1^ in G3. It demonstrates that some C-S-H gels in G3 contain Si-O-Si chains. Adding LP increases the reactants of the calcium source CaO or Ca(OH)_2_ in G3, promoting the hydration reactions of the above Formulas (2) and (3) to form more C-S-H and C-A-S-H gels. As a result, the paste G3 has a higher *UCS* at 28 d and 90 d than G2, as mentioned in Table 4.

The wavenumbers were still at 1113 cm^−1^ with the addition of GBFS, with no relative shift in G3 and G4 corresponding to the asymmetric stretching vibration of Si-O-T (Si or Al) bonds. It indicates that the product gels of C-S-H and C-A-S-H in G4 are identical to those in G3, which is consistent with the XRD patterns. Adding GBFS increases the reactants of the silica–alumina phase and the calcium source in G4, which still occur under the same hydration as the above Formulas (2) and (3). However, more gels are formed in G4 to produce higher cementation and UCS than in G3.

The wavenumber in G4 at 1113 cm^−1^ shifts to a significantly lower wavenumber in G5 at 1034 cm^−1^. The significant wavenumber shift of 79 cm^−1^ demonstrates that the addition of RM promotes the Si-O-Al chain form. In G5, geopolymeric aluminosilicate gels coexist with hydrated C-S-H and C-A-S-H gels. This is because the addition of RM raises the pH value of the mixture and provides the silica–alumina phase reactants. Under higher alkaline conditions, the higher silica–alumina phase content promotes geopolymerization, resulting in geopolymer gels (N,C)-A-S-H with Na^+^ and Ca^2+^ cations [28]. Therefore, the hydration reactions are expressed as Formulas (2) and (3) with the addition of RM, while the geopolymerization reactions are expressed as Formulas (4) and (5) [4,7,21]. The reaction parameter is *m*.

*m*(Si_2_O_5_, Al_2_O_3_) + 2*m*SiO_2_ + 4*m*H_2_O+ (Na^+^, K^+^, Ca^2+^) + OH^−^ →(Na^+^, K^+^, Ca^2+^) + *m*(OH)_3_-Si-O-Al^−^(OH)_2_-O-Si-(OH)_3_ (4)

*m*(OH)_3_-Si-O-Al^−^(OH)_2_-O-Si-(OH)_3_ + (Na^+^, K^+^, Ca^2+^) + OH^−^ →4*m*H_2_O + (Na^+^, K^+^, Ca^2+^)-[Si(OH)_2_-O-Al^−^(OH)_2_-O-Si-(OH)_3_-O-]*_m_* (5)

According to the results of the analyses, the product gels in backfill paste G5 are indeed a mixture of (N,C)-A-S-H gels with C-S-H and C-A-S-H gels, which determines the chemical cementation of backfill paste G5 using various industrial solid wastes.

Many alkali-activated mixture systems exhibit good solidification under the required curing conditions, such as the systems of FA-SR-Na_2_SiO_3_ solution [7], FA-SR-Na_2_SiO_3_ solution incorporating hemihydrate gypsum [30], and FA-CS-NaOH solution [5], as well as, especially, the systems of SR-CS-GBFS-based [8], FA-GBFS-based [31], RM-FA-based [4], CS-GBFS-based [2], and LP-GBFS-based [12], etc. As can be seen, an alkaline activation system with good solidification is mainly comprised of silica–alumina precursors, alkaline activators, and calcium sources. All alkali-activated synthesis processes occur in the blew reaction mechanism. 

Although a Ca source is not necessary for the alkaline activation system, the final products were determined by the main detected elemental components, including Na, K, Ca, O, and Al, to analyze the reaction mechanism. First, alkaline activators erode and dissolve the amorphous silica–alumina precursors, while the Si and Al tetrahedrons undergo reorganization and polycondensation to produce a network structural product [4]. The negative charge caused by the Al tetrahedron in the structure is balanced by the alkali metal cations *M*, which include Na^+^, K^+^, and Ca^2+^, resulting in an electrically neutral and stable structure [47,65]. The *M* reacts with -OSi(OH)_3_, Al(OH)_4_^−^, and other intermediate products to balance the Coulombic electrostatic repellence. Moreover, the empirical formula of the geopolymeric product is expressed as *M_n_*[-(SiO_2_)*_z_*-AlO_2_-]*_n_*, where the Si/Al ratio *z* equals 1, 2, and 3, *M* is the alkali metal cations such as Na^+^, K^+^, and Ca^2+^, etc., and *n* is the geopolymeric degree of Si and Al [4]. When the elemental components of products satisfy the relationships of 2Ca/Al < 1.0 and Si/Al > 1.0, the final gel products are composed of N-A-S-H or (N,C)-A-S-H from geopolymerization [7,47]. When the relationships of 2Ca/Al > 1.0 and Si/Al > 1.0 are satisfied in the elemental components of the products, the products are the coexistence of N-A-S-H and calcium (alumino)silicate hydrated C-(A)-S-H gels formed from the geopolymerization and hydration reactions due to the excessive Ca^2+^ as a structure-changing cation [21]. A certain amount of Ca^2+^ is required for geopolymerization with excessive Ca^2+^, while others combine with the Si monomers (and Al monomers) to form C-(A)-S-H gels. As a result, the final binder products can be determined using a combination of experimental techniques and quantitative calculations based on the geopolymerization and hydration reaction mechanisms.

According to the aforementioned fundamental synthesis compositions and synthesis mechanisms, the substitution of raw materials is easily achieved for the preparation of backfill paste. Some FA is replaced by GBFS and RM as silica–alumina precursors, and some SRs are replaced by CS and BRS as calcium sources. The additives GP and LP are used to improve the properties of the backfill paste. Strong alkaline activators, such as NaOH and Na_2_SiO_3_, however, are not recommended for use due to strong erosion in the stirring container and the exudation of alkaline OH^−^ in the environment. The cementation mechanism of new backfill paste using diverse solid wastes is revealed for the products’ mixture of C-S-H, C-A-S-H, and (N,C)-A-S-H gels along with some insoluble particles. Chemically, the soluble inorganic salts dissolve to yield reactive cations and ions such as Ca^2+^, Na^+^, [SiO_4_]^4−^, [AlO_4_]^5−^, and OH^−^, which produce cementation due to the gel form of silicate and aluminosilicate products. In the backfill paste, some insoluble inorganic salts, such as CaCO_3_, and insoluble substances, such as mullite and quartz, act physically as fine aggregates. According to the cementation mechanism, soluble cations and ions are required for the chemical bonding of backfill paste. As a result, future research should look into the effects of insoluble salts such as calcite carbonate, mullite, quartz, etc., as well as organic salts such as calcium oxalate, carbohydrate, etc., on the properties of geopolymer backfill paste. It facilitates the introduction of waste slags and wastewater-containing organic substances into geopolymer backfill paste.

## 4. Conclusions

The purpose of this paper is to assess the hardening feasibility and cementation mechanism of adopting various industrial solid wastes to make backfill paste. The main contributions based on the experimental results are presented as follows:

(1) In terms of synthetic feasibility, a new backfill paste Q1 for goaf was synthesized using a mixture of diverse solid wastes (FAI, FAII, SR, CS, GBFS, RM, and BRS) and additives (LP and GP) by directly adding an appropriate proportion of water. The backfill paste Q1, incorporating various solid wastes, exhibits good hardening with a W/B of 0.70. The used industrial solid wastes (SR, CS, GBFS, FAI, FAII, RM, and BRS) account for approximately 46.0%. The fluidity of Q1 is 201 mm, the initial setting time is 920 min, the final setting time is 1220 min, and the UCS is 2.1 MPa in 28 d UCS, which meets the property requirements of goaf backfill.
(2) In terms of cementation mechanism, FAI, FAII, GBFS, and RM provide SiO_2_ and Al_2_O_3_ precursors in alkaline conditions, while CS, SR, GBFS, BRS, LP, and GP supply calcium sources such as CaO, Ca(OH)_2_, and CaCl_2_, among others. Additionally, LP, RM, and GBFS increase the pH. It is verified that the coexistence of (N,C)-A-S-H, C-S-H, and C-A-S-H gels from geopolymerization and hydration reactions supports the chemical cementation at a high pH of 12.37, while other unreacted particles (CaCO_3_ and unreactive FA particles, etc.) act as the physical filling aggregate. As a result, the chemical cementing and physical filling work together to create the bonding structure of the new backfill paste Q1.

To summarize, it is necessary to develop a new geopolymer paste for goaf backfill by using diverse industrial solid wastes. The research results not only obtain the synthesis method but also provide information on fresh and hardened backfill paste. The outcomes have important implications for the interchange of solid waste with the same components. In the next research work, it is worthwhile to investigate the reuse of various industrial solid wastes as cementitious materials to solidify wastewater for an exploration of the broader application of new materials incorporating organic components.

## Figures and Tables

**Table 1:** The chemical compositions of raw materials measured by XRF test (mass %). LOI refers to the loss on ignition at 1000 °C.

Index	FAI	FAII	RM	CS	BRS	SR	GBFS	GP	LP	OPC
SiO_2_	53.6	46.1	27.5	8.7	14.7	9.2	35.1	3.0	0.1	21.8
Al_2_O_3_	33.5	23.2	28.4	0.5	1.2	8.7	16.2	3.6	0.0	5.7
CaO	2.8	5.4	2.5	62.6	72.6	36.4	33.6	30.5	71.2	65.3
Fe_2_O_3_	0.0	8.0	25.8	1.0	1.1	3.4	0.0	0.0	0.0	3.5
MgO	1.6	2.6	0.2	1.7	0.0	6.8	11.1	0.3	1.8	0.0
K_2_O	1.5	1.8	0.1	0.0	0.0	0.3	0.0	0.0	0.0	0.0
SO_3_	0.9	0.8	0.8	0.0	1.4	5.5	0.0	40.6	1.6	2.8
Na_2_O	0.0	0.0	14.7	0.0	0.0	3.9	0.0	0.3	0.0	0.0
P_2_O_5_	0.4	0.6	0.0	0.0	0.0	0.0	0.0	0.0	0.0	0.0
TiO_2_	1.3	0.3	0.0	0.0	0.0	0.1	0.0	0.0	0.0	0.0
Cl-	0.0	0.0	0.0	0.0	0.0	23.1	0.0	2.3	0.0	0.0
other	1.5	3.5	0.0	0.0	4.7	0.0	4.1	0.0	0.0	0.0
LOI	3.0	7.6	0.0	25.4	4.3	2.8	0.0	19.5	25.3	0.9
PH	10.24	8.30	10.52	8.55	10.07	9.32	11.45	8.25	12.86	12.87
EC	1075	2750	2180	2700	1632	3710	440	4.6	7580	8970
SSA	640	390	360	420	345	307	660	300	320	370
SG	2.48	2.25	2.56	1.80	1.10	2.35	2.67	2.98	1.10	2.89

Note: FAI = class I fly ash, FAII = class II fly ash, RM = red mud, CS = carbide slag, BRS = briquette residue slag, SR = soda residue, GBFS = granulated blast furnace slag, GP = gypsum powder, LP = lime powder, and OPC = ordinary Portland cement (#325). EC = electronical conductivity. The pH and EC were measured at an identical solid-water ratio of 1:5 by mass. PH = mean pH (−), EC = mean EC (µS/cm), SSA = specific surface area (m^2^/kg), and SG = specific gravity (−).

**Table 2:** The designed mixing proportions of backfill pastes for physical and mechanical properties, as well as microcharacterization.

No.	CS (g)	SR (g)	FAII (g)	BRS (g)	GP (g)	FAI (g)	LP (g)	GBFS (g)	RM (g)	OPC (g)	NaOH (g)	Water (g)	W/B
Q1	200	100	400	100	100	100	200	100	100	—	—	980	0.70
Q2	—	—	—	—	—	—	—	—	—	1400	—	900	0.64
Q3	200	100	400	100	100	100	200	100	100	—	200	980	0.61
G1	100	50	200	50	50	—	—	—	—	—	—	315	0.70
G2	100	50	200	50	50	50	—	—	—	—	—	350	0.70
G3	100	50	200	50	50	50	100	—	—	—	—	420	0.70
G4	100	50	200	50	50	50	100	50	—	—	—	455	0.70
G5	100	50	200	50	50	50	100	50	50	—	—	490	0.70

Note: W/B refers to the water–binder ratio. G5 is identical to Q1.

**Table 3:** The fresh and hardened performances of backfill paste and controls.

Sample No.	Fluidity (mm)	Initial Setting Time (min)	Final Setting Time (min)	28 d *UCS* (MPa)	Environmental pH (−)	Environmental EC (mS/cm)
Q1	201 (±5)	920 (±15)	1220 (±20)	2.1 (±0.1)	7.60 (±0.05)	6.50 (±0.02)
Q2	210 (±4)	770 (±20)	1540 (±25)	15.2 (±0.3)	12.30 (±0.05)	3.50 (±0.02)
Q3	185 (±5)	730 (±20)	980 (±18)	2.0 (±0.1)	13.60 (±0.05)	7.60 (±0.02)
RV [30]	≥200	≥720	≤2160	≥0.6	—	—

Note: Required value (RV) refers to the basic engineering requirement for OPC paste.

**Table 4:** The measured physical and chemical performances of pastes (G5) and controls (G1, G2, G3, and G4).

Sample No.	Temp. of Solids (°C)	Temp. of Water (°C)	Temp. of Mixture for 2 min (°C)	Fluidity (mm)	28 d UCS (MPa)	90 d UCS (MPa)	PH (−)	EC (mS/cm)
G1	28.5	27.5	29.0	170	≤0.2	≤0.2	9.24	19.18
G2	28.5	27.5	29.0	180	≤0.2	≤0.2	9.15	18.42
G3	28.5	27.5	29.0	165	1.4	1.1	12.23	18.15
G4	28.5	27.5	29.0	189	4.4	3.6	12.31	17.78
G5	28.5	27.5	29.0	198	2.1	3.4	12.37	17.12

Note: Temp. = temperature. The experimental deviations are ±0.1 °C in temp., ±3 mm in fluidity, ±0.1 MPa in *UCS*, ±0.05 in pH, and ±0.02 mS/cm in EC.

## Data Availability

Not applicable.
